# Supplementary material for: WordCommentsAnalyzer: A windows software tool for qualitative research
Source: F1000Res. 2018 Sep 4;7:536. Originally published 2018 May 3. [Version 2] doi: 10.12688/f1000research.14819.2 (PMC6137411; doi:10.12688/f1000research.14819.2)
Supplement: Supplementary file 3 [file f1000research-7-17614-s0002.tgz › b2a79496-4ada-47c4-9682-a99ce867836d.zip!/Eight Iranian Health Journals/Archives of Rehabilitation_47.docx]

© Archives of Rehabilitation. This work is licensed under CC BY-NC 4.0 (https://creativecommons.org/licenses/by-nc/4.0/).

Volume & Issue: دوره 8 - شماره 4

Title: توانایی آزمون‌های تحملی در پیش‌آگهی مزمن‌شدن علائم در بیماران مبتلا به اولین تجربه کمردرد حاد غیراختصاصی

http://rehabilitationj.uswr.ac.ir/article-1-175-fa.html

هدف: علیرغم ارزیابی تحمل عضلات تنه در بیماران دچار کمردرد مزمن، این کار در بیماران حاد یا انجام نشده و یا در بیمارانی انجام شده که چندین تجربه کمردرد حاد داشته‌اند. هدف از این تحقیق مقایسه‌ میزان تحمل عضلات فلکسور و اکستانسور تنه در بیماران مبتلا به اولین تجربه کمردرد با افراد سالم و همچنین امکان پیش‌بینی طولانی شدن علائم بوده است.   روش بررسی: در مرحله اول این تحقیق، طی یک مطالعه تحلیلی مقطعی از نوع مورد – شاهدی 30 بیمار دچار اولین تجربه کمردرد حاد که با استفاده از نمونه‌گیری غیر تصافی ساده انتخاب شده بودند، با 51 فرد سالم که بر اساس همتاسازی و جورکردن با بیماران انتخاب شده بودند، از نظر متغیرهای مورد بررسی مقایسه شدند. از آزمونهای تحمل اکستانسوری و فلکسوری ایتو برای ارزیابی تحمل عضلات تنه استفاده گردید و برای بررسی بهبودی از متغیرهای درد و ناتوانی استفاده شد.در مرحله دوم تحقیق، طی یک مطالعه تحلیلی طولی و آینده‌نگر، بیماران تا شش هفته پیگیری شده و به دو گروه بهبودیافته و بهبود نیافته تقسیم شده و توانایی آزمونهای انجام شده بر روی آنان، در پیش آگهی بهبودی یا مزمن شدن درد در آنان بررسی گردید. جهت تجزیه و تحلیل داده‌ها از آزمونهای آماری کولموگراف–اسمیرنوف، تی مستقل و مجذور خی استفاده شد.   یافته‌ها: نتایج آزمونهای تی مستقل نشان دهنده تحمل عضلانی پایین‌تر اکستانسوری و فلکسوری در گروه بیماران نسبت به افراد سالم بود (P<0/0001). بعلاوه تحمل فلکسوری بیمارانی که پس از شش هفته بهبود نیافتند، از ابتدا کمتر از بیماران بهبود یافته بود (P<0/05).   نتیجه گیری: به نظر می‌رسد که بیماران کمردرد در اولین تجربه خود دچار کاهش تحمل شده و برای کاهش تحمل نیاز به تکرار دفعات حمله نباشد. همچنین آزمونهای تحملی می‌توانند در پیش‌آگهی ناتوانی و مزمن شدن کمردرد و تفکیک بیمارانی که در معرض این رخداد هستند، بعنوان روشی غیر تهاجمی و کم هزینه نقش ارزنده‌ای داشته باشد.

Title: تأثیر ماساژ به روش استروک سطحی پشت بر اضطراب سالمندان

http://rehabilitationj.uswr.ac.ir/article-1-176-fa.html

هدف: اضطراب در زندگی امروزی، به خصوص در سالمندان که قدرت تطابق و سازگاری کمتری دارند، می‌تواند باعث عوارض وخیم و جبران ناپذیری در ابعاد سلامت آنان شود. این مطالعه با هدف کاهش میزان اضطراب آشکار این افراد با استفاده از تکنیک ماساژ استروک سطحی پشت «اس.اس.بی.ام» در سالمندان انجام شد.   روش بررسی: این مطالعه به روش نیمه تجربی در مرکز سالمندان کهریزک تهران در سال 1385 انجام شد. 102 نفر سالمند بر اساس معیارهای ورودی انتخاب و با روش بلوک‌های تبدیل شده تصادفی به دو گروه مساوی مداخله و کنترل تخصیص داده شدند. ابزار گردآوری داده‌ها در این پژوهش شامل فرم مشخصات دموگرافیک و پرسشنامه اضطراب آشکار اسپیل برگر بود. در گروه مداخله ماساژ «اس.اس.بی.ام» به مدت 5 روز متوالی، روزی 5 دقیقه بین ساعات 7-3 بعد ظهر اجرا گردید. میزان اضطراب آشکار هر دو گروه قبل و بعد از مداخله مورد ارزیابی قرار گرفت. از آزمون‌های کای اسکوئر، تی مستقل، کولموگروف- اسمیرنوف و تی زوجی جهت تجزیه و تحلیل اطلاعات استفاده شد.   یافته‌ها: میانگین اضطراب آشکار دو گروه مداخله و کنترل قبل از شروع ماساژ «اس.اس.بی.ام» اختلاف معناداری نداشت (P>0/05)، در حالی که بعد از ماساژ « اس.اس.بی.ام» اختلاف معناداری بین دو گروه مشاهده شد (P<0/05).   نتیجه‌گیری: با توجه به نتایج مطالعه می‌توان چنین اظهار نمود که با بکارگیری روش ماساژ « اس.اس.بی.ام» امکان کاهش اضطراب و عوارض ناشی از آن در سالمندان وجود دارد.

Title: مقایسه‌ تأثیر تحریکات حسی برسطح هوشیاری بیماران دچار اغما در دو زمان دیرهنگام و زودهنگام

http://rehabilitationj.uswr.ac.ir/article-1-177-fa.html

هدف: امروزه بر توانبخشی زودهنگام و صحیح بیماران دارای اختلال هوشیاری تأکید فراوان دارند، اما تحقیقات انجام شده در این زمینه حاکی از ارائه این مداخلات در زمان‌های متفاوت می‌باشد. لذا به منظور مقایسه میزان افزایش سطح هوشیاری متعاقب تحریکات حسی در دو زمان زودهنگام و دیرهنگام مطالعه حاضر انجام شد. روش بررسی: در یک مطالعه تجربی و مداخله‌ای و به طریق سنجش‌های مکرر، 21 مرد دچار اغما که GCS آنها در 3 روز اول پس از ضربه، 8 یا پایین‌تر بود، به صورت تصادفی به 3 گروه مقایسه، مداخله زودهنگام و مداخله دیرهنگام تخصیص یافتند. تمامی‌بیماران تا روز 25 پس از ضربه پیگیری شده و سطح هوشیاری آنان با دو معیارGCS و CRS-R به صورت یکروز درمیان ارزیابی شد. مداخله تحریک حسی یک هفته‌ای با تحریک 5 حس شنوایی، ‌بینایی، لامسه، حرکتی و بویایی در‌گروه زودهنگام از روز 7-5 پس از ضربه و در گروه دیرهنگام از روز 15 پس از ضربه ارائه شد. همچنین طی هفته انجام مداخله، روزانه قبل و بعد از دریافت مداخله سطح هوشیاری ارزیابی شد. داده‌‌های بدست آمده با استفاده از آزمونهای آماری تی زوجی، همبستگی پیرسون و اندازه‌گیریهای مکرر مورد تجزیه و تحلیل قرار گرفت. یافته‌ها: تحلیل روند تغییرات سطح هوشیاری در طول 25 روز اول پس از ضربه حاکی از افزایش معنادار نمره دو معیار نامبرده در هر 3 گروه بود (P<0/001). مقایسه میزان تغییرات نمره GCS در 3 گروه توسط آزمون تعقیبی post hoc تفاوت معناداری بین 3 گروه نشان نداد (0/15=P). اما میزان تغییرات نمره CRS-R در دو گروه زودهنگام و دیرهنگام تفاوت معناداری را نشان داد(P<0/05). نتیجه‌گیری: بیداری بیماران اغمایی در طول 25 روز اول پس از ضربه در هر 3 گروه بهبود خودبخودی دارد. همچنین یافته‌های تحقیق حاکی از آن است که ارائه تحریکات حسی زودهنگام می‌تواند این روند بهبود خودبخودی را تسریع کند.

Title: تأثیر تزریق سلول‌های استرومایی مغز استخوان در بهبود اثرات صرع ناشی از تزریق پیلوکارپین در موش صحرایی

http://rehabilitationj.uswr.ac.ir/article-1-178-fa.html

هدف: صرع با منشاء لوب گیجگاهی رایج ترین نوع صرع در انسان است و مهمترین مشخصه آن بروز حملات تکرار شونده در بیمار است. با توجه به عوارض ناشی از داروهای ضد صرع و خطرات ناشی از جراحی‌ها، در این تحقیق تأثیرات ناشی از تزریق سلول‌های استرومایی مغز استخوان در موش‌های صحرایی صرعی مدل پیلوکارپین مورد ارزیابی قرار گرفت . روش بررسی: در این تحقیق تجربی موش‌های صحرایی بطور تصادفی به 5 گروه 5 تایی تقسیم شدند: گروه کنترل (درمان نشده)، سه گروه درمان شده با تزریق 2 الی 3 میلیون سلول‌های استرومایی مغز استخوان نشان دار شده با BrdU بعد از 12، 24 و 36 ساعت از شروع اولین حمله صرع و یک گروه به عنوان گروه شم که تنها نرمال سالین دریافت کردند. برای ارزیابی رفتاری، موش‌های صحرایی به مدت 3 هفته در مرحله مزمن بیماری به صورت شبانه‌روزی مانیتورینگ شدند و برای این منظور از مقیاس Racine استفاده شد. شش هفته بعد از شروع اولین حمله موش‌های صحرایی کشته شدند و مغز آنها با روش پارافینی و کرایو مورد پردازش بافتی قرار گرفت. داده‌های حاصل با استفاده از آزمونهای آماری ویلکاکسون و توکی مورد تجزیه و تحلیل قرار گرفت. یافته‌ها: میانگین تعداد حملات در طی 3 هفته برای گروه کنترل 6/25±1/3، گروه شم 6/2±0/8، گروه درمان شده با سلول‌های استرومایی مغز استخوان بعداز 24 و 36 ساعت از اولین حمله به ترتیب 2±0/4 و 2/25±0/47 بدست آمد و در گروه 12 ساعته همه موش‌های صحرایی مردند. بین تعداد حملات در گروههای 24 و 36 ساعته با سایر گروهها تفاوت معنی‌داری وجود داشت (P<0/01). همچنین تعداد سلولها در بافتهای مورد بررسی در گروههای درمان شده (24 و 36 ساعته) نسبت به سایر گروهها افزایش معناداری نشان داد (P<0/01). نتیجه‌گیری: تزریق وریدی سلول‌های استرومایی مغز استخوان در کاهش حملات ناشی از صرع مدل پیلوکارپین مؤثر است. همچنین موجب جلوگیری از آسیب‌های دژنراتیو بافتی و کاهش سلولها ناشی از ایجاد تشنج می‌شود.

Title: مقایسه توانایی‌های تعادلی در بیماران مبتلا به فلج نیمه راست یا چپ بدن ناشی از سکته مغزی

http://rehabilitationj.uswr.ac.ir/article-1-179-fa.html

هدف: ارتقاء سطح استقلال فردی بیماران فلج نیمه بدن، بخصوص در حفظ تعادل از اهداف عمده توانبخشی می‌باشد. با توجه به اینکه محل ضایعه مغزی در بیماران همی پلژی راست و چپ و همچنین عملکرد نیمکره‌های راست و چپ مغز متفاوت می‌باشد، هدف این مطالعه مقایسه توانایی‌های تعادلی در این دو گروه از بیماران بوده است. روش بررسی: این مطالعه از نوع مقطعی مقایسه‌ای و بر روی 30 بیمار مبتلا به سکته مغزی که به بیمارستان بقیه‌الله(عج) مراجعه کرده و شامل گروه بیماران فلج نیمه راست بدن (16 بیمار) و گروه بیماران فلج نیمه چپ بدن (14بیمار) بودند انجام شد. محدوده سنی بیماران 58-30 سال بوده و مدت 6 ماه از زمان سکته آنها می‌گذشت. نمونه‌ها از موارد در دسترس و به صورت هدفمند و ساده انتخاب و در هر دو گروه، توانایی‌های تعادلی افراد با استفاده از دو آزمون بالینی مقیاس تعادلی برگ (B.B.S) و آزمون مایل شدن به جلو (F.F.R) ارزیابی و اطلاعات بدست آمده با استفاده از آزمونهای آماری کولموگراف-اسمیرنوف، تی مستقل و تی زوجی مورد تجزیه و تحلیل آماری قرار گرفت. یافته‌ها: اختلاف میانگین نمره کسب شده در آزمون B.B.S توسط دو گروه معنی‌دار بوده (P<0/05)، و میانگین نمرات گروه همی پلژی راست (38/36) بیشتر از میانگین نمرات گروه همی پلژی چپ (28/90) بود، درصورتیکه در آزمون F.F.R اختلاف میانگین نمرات دوگروه با همدیگر معنی‌دار نمی‌باشد (P=0/841). نتیجه‌گیری: مطابق نتایج بدست آمده، تعادل خصوصاً از نوع داینامیک آن با سمت درگیر رابطه مستقیم دارد، درحالیکه در تعادل استاتیک چنین چیزی صادق نمی‌باشد و می‌توان مطابق نتایج این تحقیق برنامه توان بخشی مناسبی را جهت بهبود تعادل این بیماران در نظر گرفت.

Title: مقایسه وضعیت قرارگیری استخوان کتف در دو اندام غالب و غیرغالب در دختران دانشجو

http://rehabilitationj.uswr.ac.ir/article-1-180-fa.html

هدف: این تحقیق با هدف مقایسه وضعیت قرارگیری استخوان کتف (پروترکشن، چرخش) و میزان لغزش جانبی کتف در هنگام بالا بردن بازو در دو سمت غالب و غیر غالب در دختران جوان انجام شد. روش بررسی: به روش نمونه‌گیری غیر احتمالی و ساده، 30 دختر با محدوده سنی 25-20 سال در این مطالعه مقطعی مقایسه‌ای شرکت نمودند. برای اندازه‌گیری وضعیت استراحت کتف (پروترکشن و چرخش کتف) از روش دایویتا و برای سنجش عدم تقارن کتف از تست لغزش جانبی کیبلر استفاده گردید. به جهت بررسی تکرارپذیری و پایایی روشهای اندازه‌گیری از تعیین ICC و SEM و به منظور تجزیه و تحلیل داده‌‌های حاصل از اندازه‌گیری‌ها، از آزمون‌ آماری تی زوجی استفاده شد. یافته‌ها: متغیرهای پروترکشن و چرخش در دو سمت غالب و غیر غالب اختلاف معنی‌داری را نشان نداد (P=0/61 و P=0/57). اختلاف معنی‌داری بین لغزش جانبی کتف در وضعیتی که دستها در کنار بدن قرار داشت در دو سمت غالب و غیرغالب مشاهده نگردید (P=0/66)، درحالیکه در وضعیتی که دستها روی کرست‌های ایلیاک و ابداکشن 90 درجه همراه با چرخش داخلی قرار داشت، کتف در سمت غالب بطور معنی‌داری خارج‌تر نسبت به سمت غیر‌غالب قرار داشت (0/001>P). نتیجه‌گیری: غالب بودن دست مسئول درجاتی از عدم تقارن در وضعیت قرار گیری کتف‌ها می‌باشد.

Title: ارزیابی دست‌نویسی دانش‌آموزان کم‌توان ذهنی: معرفی یک ابزار

http://rehabilitationj.uswr.ac.ir/article-1-181-fa.html

هدف: یکی از مشکلات آموزشی دانش‌آموزان کم‌توان ذهنی در مدارس استثنایی اختلالات دست‌نویسی است. به منظور ارزیابی بهتر این اختلالات، این پژوهش با هدف ساخت ابزاری برای ارزیابی دست‌نویسی و اجرای آن بر روی دانش‌آموزان کم‌توان ذهنی انجام گرفت. روش بررسی: در این پژوهش توصیفی و مقطعی که از نوع مطالعات همبستگی و بخشی از آن نیز اعتبارسنجی ابزار است، از 157 دانش‌آموز کم‌توان ذهنی دختر و پسر که در سال تحصیلی 85-1384 در پایه‌های اول تا پنجم مدارس استثنایی شهرری مشغول به تحصیل بودند، پس از حذف نمونه‌هایی که معیارهای خروج از مطالعه را شامل بودند، مابقی 126 دانش‌آموز کم‌توان ذهنی (53 دختر و 73 پسر) که در دامنه سنی 9 تا 19 سال قرار داشتند، به روش تمام‌شمار شرکت نمودند. برای همه دانش‌آموزان چک لیست دست‌نویسی محقق ساخته اجرا شد. داده‌‌های بدست آمده با آزمونهای آماری تی مستقل، یومن و یتنی و همبستگی اسپیرمن مورد تجزیه وتحلیل قرار گرفتند. یافته‌ها: نتایج بدست آمده نشان داد که دست‌نویسی با جانبی شدن (P=0/196)، جنسیت (P=0/487) و با سن (P=0/449) رابطه معنی‌دار ندارد. اما با پایه تحصیلی (r=0/448, P<0/005) رابطه معنی‌دار دارد، که البته بین پایه‌های مختلف تفاوت معنادار مشاهده نشد. شایع ترین مشکل دست‌نویسی در دانش‌آموزان کم‌توان ذهنی شهرری جدانویسی با 82 درصد و کم‌اهمیت ترین مشکل دست‌نویسی آنان، فاصله گذاری بین کلمات و حروف با 34/9 درصد بود. نتیجه‌گیری: فراوانی اختلالات مربوط به اجزای خوانایی دست‌نویسی در دانش‌آموزان کم‌توان ذهنی مبین این امر است که اختلالات دست‌نویسی در مدارس استثنایی نیازمند توجه تخصصی است.

Title: سنجش میزان رضامندی مددجویان از خدمات توان‌بخشی جمعیت هلال احمر شهر تهران

http://rehabilitationj.uswr.ac.ir/article-1-182-fa.html

هدف: این مطالعه، با هدف سنجش میزان رضامندی مددجویان از خدمات توانبخشی در مراکز توانبخشی سازمان هلال احمر شهر تهران انجام شده است. این تحقیق تلاش دارد سازمان را در دستیابی به نقاط قوت وضعف خود یاری داده و به ارتقاء کیفیت و کارایی خدمات توانبخشی کمک کند. روش بررسی: این مطالعه به روش توصیفی و پیمایشی انجام شده است. جمع‌آوری اطلاعات با استفاده از پرسشنامه محقق‌ساخته صورت گرفته و پایایی آن 82/0 محاسبه شده است. تعداد 190 نفر از مددجویان مراکز توانبخشی جمعیت هلال احمر شهر تهران با استفاده از سیستم طبقه‌بندی و خوشه‌ای انتخاب شده و در این مطالعه شرکت کردند. داده‌های حاصل با استفاده از آزمونهای آماری خی دو، تی مستقل و آنالیز واریانس مورد تجزیه و تحلیل قرار گرفت. یافته‌ها: میزان مشارکت در فرایند درمان P=0/021)، کارایی وسیله توانبخشی (P=0/002)، کیفیت خدمات توانبخشی (P<0/001) و رفتار و برخورد پرسنل (P<0/001) همگی با رضامندی مددجو ارتباطی معنادار داشت. نتیجه‌گیری: نتایج این تحقیق مبین این نکته است که خدمات توانبخشی، تحت تأثیر عوامل مختلف منجر به کاهش رضامندی مددجویان شده است. رضامندی را می‌توان تابعی از میزان مشارکت مددجو در فرایند درمان، میزان کارایی و کیفیت خدمات و نوع رفتار وبرخورد پرسنل (پزشکان، تکنسین‌ها و...) تلقی کرد. لذا با برگزاری دوره‌های آموزشی کوتاه مدت برای پرسنل، تجهیز مراکز به امکانات مناسب توانبخشی و آگاه‌سازی و فراهم کردن امکان مشارکت مددجو درفرایند درمان می‌توان انتظارات مددجویان و خانواده‌های آنان را برآورده ساخته و رضامندی آنها را افزایش داد.

Title: تعیین مشخصات روان سنجی مقیاس درجه‌بندی SNAP-IV، اجرای والدین

http://rehabilitationj.uswr.ac.ir/article-1-183-fa.html

هدف: پرسشنامه SNAP-IV، مقیاسی جهت تشخیص و درجه‌بندی اختلال بیش‌فعالی – کمبود توجه است که توسط سوانسون، نولان ‌و پلهام تألیف شده است. هدف از این پژوهش تعیین مشخصات روان سنجی این آزمون در جامعه کودکان 7 تا 12 سال شهر تهران بر اساس نظرات والدین است. روش بررسی: پژوهش توصیفی حاضر از نوع مطالعات متدولوژیک، کاربردی و اعتبارسنجی است. از جامعه کودکان تهرانی، نمونه‌ای به حجم 1000 نفر به روش نمونه‌گیری خوشه‌ای تصادفی انتخاب شدند و سپس از مادران این کودکان خواسته شد طبق مقیاس SNAP-IV، کودک خود را ارزیابی کنند. 686 پرسشنامه بطور کامل پاسخ داده شد و مابقی بعلل مختلف و نقص‌های موجود حذف گردید. بدین ترتیب حجم نمونه‌ نهایی 686 نفر بود. 30 نفر از اعضاء نمونه مجدداً یک ماه پس از اجرای اصلی، مورد آزمون قرارگرفتند. همچنین مصاحبه بالینی نیز روی 30 نفر از نمونه‌ها انجام شد. جهت بررسی و تحلیل داده‌‌ها از آزمونهای آماری ضریب همبستگی پیرسون، کولموگراف – اسمیرنوف، لوین و آزمون تی برنز فیشر استفاده شد. یافته‌ها: روایی ملاکی آزمون 0/48 و طبق تحلیل عامل این آزمون دارای 3 عامل است که مجموعاً %56 واریانس را تبیین می‌کنند. روایی محتوا نیز مورد تأیید متخصصان است. ضریب پایایی بازآزمون برابر با %82، آلفای کرونباخ 0/90 و ضریب دو نیمه کردن 0/76 می‌باشد. نقطه برش در کل مقیاس و هر کدام از خرده مقیاس‌‌های کمبود توجه و بیش فعالی به ترتیب برابر با 1/57 و 1/45 و 1/9 است. نتیجه‌گیری: یافته‌های پژوهش نشان می‌دهد آزمون از مشخصات روان سنجی مناسبی برخوردار است و استفاده همراه با اطمینان آن را در موقعیت‌های مختلف پژوهشی، تشخیصی و درمانی توصیه می‌کند.

Title: مقایسه حس نیروی استاتیک اکستانسوری زانو در زنان مبتلا به درد مفصل کشککی-رانی و زنان سالم

http://rehabilitationj.uswr.ac.ir/article-1-184-fa.html

هدف: سندرم درد مفصل کشککی- رانی، یکی از شایعترین علل درد سیستم عضلانی اسکلتی است. حس عمقی یا آگاهی از حرکت و وضعیت مفصل، جزء مهم برنامه‌های کنترل حرکت و تعادل بوده ومطالعات گذشته نشان داده است که این حس در بیماران مبتلا به درد مفصل کشککی-رانی دستخوش آسیب و تغییر می‌شود. هدف مطالعه حاضر مقایسه دقت حس نیروی استاتیک عضله چهار سر رانی در بیماران مبتلا به درد قدامی زانو و افراد سالم می‌باشد. روش بررسی: مطالعه تحلیلی – قطعی انجام شده از نوع مورد-شاهدی بوده و 17 زن مبتلا به درد قدامی زانو با انتخاب هدفمند و غیر احتمالی و 17 زن سالم بر اساس همتاسازی و جور کردن با بیماران در آن شرکت کردند. برای ارزیابی دقت حس نیروی استاتیک عضله چهار سررانی در دو گروه، از دستگاه دینامومتر ایزوکینتیک بایودکس و از روش برآورد نیرو با اندام تحتانی یک سمت بدن، استفاده شد. بدین صورت که افراد با ترتیب تصادفی در یکی از زوایای 20 تا 60 درجه خمیده زانو، بزرگی نیروهای 20 تا 60 درصد حداکثر نیروی عضلانی عضله چهارسررانی را تولید کرده و پس از 5 ثانیه و بر اساس میزان درک نیروی داخل عضله، نیروی مورد نظر را برآورد و بازسازی می‌کردند. برای اندازه‌گیری دقت برآورد نیروهای مورد نظر خطای ثابت، مطلق و متغیر محاسبه شد. نتایج با آزمونهای آماری تی مستقل و رگرسیون لوجستیک مورد تحلیل قرار گرفت. یافته‌ها: دقت برآورد نیروی بزرگ (60 درصد حداکثر انقباض استاتیک اکستانسوری) در زاویه 60 درجه، در دو گروه اختلاف معنی‌دار آماری داشته و در گروه بیماران کمتر از افراد سالم بوده است (P<0/05). نتیجه‌گیری: مطابق با یافته‌های این مطالعه حس عمقی مفصل زانو در بیماران مبتلا به درد مفصل کشککی - رانی ممکن است به دنبال تغییرآوران‌های مربوط به گیرنده‌های عضلانی، از جمله عضله چهارسررانی، دستخوش آسیب شود.
